# Collectanea

**Published:** 1829-06

**Authors:** 


### COLLECTANEA.

Floriferis ut apes in saltibus omnia libant,
Omnia nos, itidem, depascimur aurea dicta.

### PHYSIOLOGY.

*Royal Society.—Observations relating to the Function of Digestion.* By A. P. W. PHILIP, M.D. F.R.S. &c.

The author, referring to his former papers, published in the Philosophical Transactions, concludes that digestion requires, for its due performance, both a proper supply of gastric secretion and a certain muscular action in the stomach; the latter circumstance being needful for the expulsion of that portion of food which has been acted upon by the gastric juice. Nervous power is necessary for secretion; but the muscular action of the stomach, being excited by the mechanical stimulus of the contents of that organ, is independent of the nervous power. It had already been shown by the author, that, after the removal of a portion of the eighth pair of nerves, the galvanic influence directed through these nerves will restore the secretion of gastric juice. But Messrs. BRESCHET and H. MILNE EDWARDS have lately endeavoured to prove that the same effect results also from mechanical irritation of the lower portions of the divided nerves. The author points out several circumstances which appear to have been overlooked by these gentlemen, and which he thinks invalidate the conclusions they have deduced from their experiments. He states that a certain quantity of digested food will always be found in the stomach of the animal for five or six hours after the operation, and even after the lapse of ten or twelve hours, from its being less completely changed, and therefore expelled more slowly than in the natural state. The paper concluded with the recital of experiments made for the author by Mr. CUTLER, in which the contents of the stomach of a rabbit, whose eighth pair of nerves, after excision, had been kept mechanically irritated, were compared with those of another rabbit in which the nerves had not been irritated, and of a third which had been left undisturbed. All those who witnessed the result of this experiment, among whom was Mr. BRODIE, were convinced that the irritation of the nerves had no effect whatever in promoting the digestion of the food; neither did it at all contribute to relieve the difficulty of breathing consequent upon the section of the nerves.—*Philos. Mag.*

### PATHOLOGY.

*Affection of the Heart. Extraordinary Growth.*—Dr. BEDOR has lately recorded this singular case: Dr. B. was requested to see a young man whose situation was considered by his medical attendant to be very alarming. The symptoms denoted an organic disease of the heart and great vessels, and he was evidently in a very precarious state. He had been measured three months before, and was then five feet three inches in height. Twenty-five days after, he was again measured, and he had increased in stature full three inches. A few days after the visit of Dr. B. the patient died suddenly. He was scarcely nineteen years of age, and had attained the unusual growth of six feet three inches at the time of his decease.—*La Clinique.*

*Malformations of the Heart.*—Dr. F. RAMSBOTHAM lately presented to the Hunterian Society some specimens of malformation of the heart. In the first instance, both the aorta and pulmonary artery arose from the right ventricle, there being a communication between the ventricles by an orifice in the septum. There was no appearance of lividity in the child, although a mixture of the blood must have existed. The child died suddenly at ten years of age. She was healthy until two years old, when exertion caused some shortness of breathing. She was of a lively and placid disposition. The pulse was rapid and small; the pulsations of the carotids could be distinctly observed. She lay on her back or on the left side, with the legs drawn up to the body, and the back bent to a semicircle. Appetite and general health were good. Her lips and fingers were occasionally of a bluish gray colour. The sensation to the hand applied over the heart was that of sawing, and to the ear that of the purring of a cat. Twelve months ago she became much emaciated. The appetite was greater than natural; the skin dry; the heart laboured in its action; and the respiration was much hurried, and was attended with a short irritating cough, without expectoration. Lips usually pale, but occasionally livid, and she was unable to lie down. During some months the symptoms varied in severity, and at length the face and lower extremities became anasarcous. She expired suddenly about twelve o'clock at night, jumping up as if she had been frightened.

*Dissection.*—The chest only was permitted to be opened. Half a pint of serum was found in the pericardium, and a pint and a half of bloody serum in the left cavity of the chest. There were a few old adhesions between the pleura on this side. On the right side there were no adhesions, and very little serum. The heart was much gorged with fluid blood, and had the peculiar arrangement of vessels above described.

In the second specimen Dr. R. exhibited, there was no pulmonary artery: its deficiency appeared to be supplied by the bronchial vessels. The aorta, springing from a point between the two ventricles, ran on the right side of the trachea. There were two right bronchial arteries, and one left, all enlarged. The face was very livid. The girl died at sixteen, of tubercular phthisis.

The third was an example of a single heart, one auricle and one ventricle. The heart was turned to the right side, and the ductus arteriosus seemed to supply the place of the pulmonary artery. The pulmonary veins of the left lung terminated in the left subclavian vein; that of the right lung passed through the diaphragm, and terminated in the vena portarum. There appeared an attempt to form a pulmonary artery and vein. The child died when six months old.

---

*Rupture of the Heart; Aneurismal Tumor of that Organ.* By Dr. BIGNARDI. (*Annali Univ. di Med.* Jan. 1829.)

Marianne Prezzi, æt. fifty-eight, of a lymphatic temperament and slender form, had been occasionally subject to diarrhœa. She had otherwise enjoyed good health, when, in February 1828, she complained of a burning sensation in both eyes, which prevented her from working. By appropriate treatment she was relieved in a few days. At the commencement of March she was attacked with fever, and erysipelatous inflammation at the lower part of the neck on the left side. On the 8th, she consulted Dr. B., but refused to submit to bleeding, which he recommended. She was confined to strict diet, and was ordered mild drinks. On the tenth day, that part of the neck which had been originally the seat of inflammation presented a sort of beating similar to arterial pulsation. No explanation could be given of this unusual phenomenon. The pulsations of the heart were regular, and did not correspond with those of the surface, which were not more than fifty in the minute. By bleeding and emollient applications, these symptoms were relieved, and she resumed her usual occupations.

On the 14th March, she complained of transient sensations of coldness and numbness in both hands, particularly the left. She passed a tranquil night; and on the morning of the 15th, while in the act of making her bed, she suddenly cried out that she had a giddiness of the head, and fell dead upon the spot.

*Dissection, fifty hours after death.*—The external appearance of the body presented nothing remarkable. The vessels of the brain were empty. The substance of the brain firmer than usual. Lungs very healthy and crepitating. Pericardium enormously distended with a large quantity of blood. The heart was soft and slightly discoloured: at its posterior surface, about one inch from the apex, and a few lines from the right and inferior margin, in a part not covered by fat, was found a longitudinal aperture, penetrating into the cavity of the left ventricle; it was about six lines in length and about half a line in breadth, in the direction of the longitudinal axis of the heart. The edges of the aperture were irregular and lacerated. Towards the left of this opening another fissure was found in a similar direction, covered by the pericardium. The muscular tissue of the heart surrounding both these openings was preternaturally soft. The cavities of the heart contained no blood, and were not perceptibly dilated. The parietes of the left ventricle were somewhat thickened, except at the part where the rupture had occurred. There the muscular tissue appeared to be diminished in its substance; it was soft, pale, yellow, and did not present the fibrous appearance which is natural to it. Examined with a microscope, the torn surface appeared granulated and formed of globules in the midst of a thick serosity. There was no appearance of puriform matter. All the arterial openings were in their natural state. The vena azygos was rather larger than common. The other viscera of the thorax and abdomen were healthy.

Dr. Bignardi is of opinion that the partial or total dilatation of the arteries always results from a softening of their middle coat. He conceives that the rupture which most frequently happens, and which precedes the formation of an aneurismal sac, is invariably consecutive to a morbid alteration of the middle or fibrous coat of the artery. To point out more clearly the analogy which he presumes to exist between such an alteration of the arteries and that which gives rise to rupture of the heart, he relates the following

Case.—In the winter of 1823, a young person of Modena, named Radichi, died suddenly while dressing herself for a ball. Upon dissection, the pericardium was found greatly distended with blood. Upon attentively examining the heart, there was found at the base of the left ventricle, near the origin of the aorta, a small tumor, the size of a bean. Near this tumor the laceration had taken place, which permitted the effusion of blood into the pericardium, and caused the death of the patient.

*Observations relative to the Question, " Is the appearance of the Blood abstracted as a remedial means a just criterion in considering the propriety of repeating the operation of Bloodletting?"* By John Davy, M.D. F.R.S. Physician to the Forces. (*Edinb. Med. and Surg. Journal*, April 1829. Condensed.)

Dr. Davy first considers the appearances and qualities of the blood, supposed to indicate inflammation, and to warrant rather than forbid further blood letting. They are, an unusual degree of fluidity of the blood the instant it is drawn; unusual slowness in coagulation, and, when coagulated, being covered with a buffy coat and cupped. These appearances and qualities of the blood are met with in most cases of local inflammation, but with shades, differences, and exceptions, involving much difficulty. When inflammation is violent, rapidly running on to suppuration, extensive, and attacking at the same time one or more textures in different organs, the blood drawn is often neither buffed nor cupped. Dr. D. has witnessed this in peritoneal inflammation, either pure or complicated with inflammation of the mucous coat of the intestines, or with diffuse cellular inflammation. In diffuse cellular inflammation, the blood often coagulates rapidly as in health, and yet, being unusually liquid, exhibits a slight buffy coat, if the vessel is quickly filled and remains unmoved. In ordinary cases of inflammation, as of the pleura and lungs, blood drawn at the commencement of the disease is occasionally not buffed nor cupped. The next day, on repeating the operation, the blood exhibits both these qualities. In inflammation of the mucous coat of the air-passages or alimentary canal, blood drawn may or may not exhibit the appearances and qualities mentioned.

It does not appear that there is any relation in point of degree between these appearances and qualities, and the intensity of the inflammation. Often in fatal cases, fibrinous concretions or polypi, corresponding to the buffy coat in blood drawn, are found in the heart and great vessels, and probably as often when the lancet has been used either freely, moderately, or not at all.

Dr. Davy, therefore, cannot confide in these appearances as indications for practice and the repetition of bloodletting. He concludes they are neither just nor safe criteria.

Dr. D. next considers those appearances of the blood supposed to be connected with a state of the system, as it were, the opposite of inflammation, and not to warrant further bloodletting. They are chiefly, as it is supposed, a very soft crassamentum, very little, if at all, contracted; or the blood remaining liquid, or the proportion of the crassamentum to the serum being unusually small. As far as Dr. Davy can judge, these appearances and qualities of the blood are not proved to be connected with the state of the system supposed. In remittent fever of hot climates, in cholera morbus, both the common and epidemic, the crassamentum is generally softer than natural, and little, if at all, contracted; and yet in these diseases bloodletting is not generally injurious. It is often useful, even if repeated.

Blood without fibrin in any disease is very uncommon. Dr. D. has witnessed it only in pulmonary apoplexy, and that after death in the cavities of the heart and vessels; but so soon after death, that it may be taken for granted it was not a post-mortem change. In such cases, if the patients were robust and plethoric, blood would be taken, it is presumed, without hesitation.

In the advanced stage of acute diseases, or in acute diseases supervening on chronic of long duration, or in persons of delicate habits, the proportion of crassamentum to the serum is often small. In such cases the lancet would not be used unless it were considered urgently necessary; and, if necessary, the small proportion of crassamentum it might yield would not prevent venesection.

The blood, as far as experience informs us, is not apparently altered in the continued fever of summer, in the early stages of synochus, in apoplexy and tetanus, and many diseases strictly belonging to the neuroses, in the treatment of which bloodletting is often useful, and sometimes necessary. All these considerations tend to support the former conclusion.

It appears to Dr. Davy that, the more experience we have, the less confidence we place in the appearances of the blood, either as to the nature or treatment of disease. Dr. HEBERDEN says, "the more we know of the human body, the more reason we find to believe that the seat of diseases is not to be sought for in the blood, to the sensible qualities of which they seem to have very little relation; and though it be supposed to hold in all maladies, yet in reality it is but in very few that the blood affords the practitioner any useful information." Dr. SCUDAMORE calls this a remarkable passage. Dr. Davy is of opinion that, in the state of knowledge of the blood at the time it was written, the practical conclusion was correct. He fears and believes it is still so, though our knowledge has increased; and that it is so, even more strictly than expressed, even in all diseases, and without the exception of "very few."* Dr. Davy is not aware that there is "any one disease" in which a skilful practitioner feels it necessary to examine the blood, either for the purpose of ascertaining its nature or to direct its treatment. In pneumonia and pleurisy we usually examine the blood. If it is *not* buffed, and the symptoms indicate the necessity of further depletion, we still bleed. Suppose blood abstracted in pulmonary inflammation, supervening on tubercular phthisis, is *strongly buffed*, and the symptoms are little, if at all, mitigated, shall we, on account of the state of the blood, repeat the bloodletting? Probably not. The peculiar state of the patient will rather be considered, and endeavours made to subdue the inflammation by other means.

---

*Case of Delirium Tremens, treated by local Bloodletting and Purgatives previous to the administration of Opium.* By A. H. RENTON, M.D. (*Ibid.*)

On the 21st October, 1828, at nine A.M., I was desired to see M. de P—, who, by his son's account, had fainted early in the morning, after a copious evacuation of the bowels, and had since been the subject of frequent fits of convulsions. His general habits are extremely intemperate.

I found the features puffy and bloated; the eye wild and suspicious, and the pupils contracted, and impatient of vivid light. There was general tremor; and the body, but particularly about the face and forehead, was bathed in cold perspiration. The tongue was loaded, tremulous; and the pulse 120, small and compressible. In answering my questions, his manner was agitated, but tolerably coherent. To his wife and the people of the house his conduct was quite outrageous.

On my being about to leave the room, he became suddenly insensible, and

* Mr. Thackrah, who has paid much attention to the subject of the various alterations in the appearances and qualities of the blood in different diseases and conditions of the system, is opposed to this opinion. Vide Thackrah on the Blood, p. 90-97.—EDITORS.

was almost immediately seized with a paroxysm of general convulsion, attended by foaming at the mouth and some degree of stertor, and ending in a state of stupor.

Temporibus statim applicentur Hirudines viginti, et nuchæ vesicatorium amplum. Sumat Calomel. gr. iv., Jalapæ gr. xv.; et vespere, si opus sit, injiciatur Enema domesticum.

22d, ten A.M.—The paroxysms have been less frequent, but the delirium continues unabated. He complains only of headach. The medicine has produced no effect upon the bowels.

Habeat, quam primum, Extracti Cathartici gr. v.; Olei Crotonis gtt. dimidiam; et repet. dosis quarta quaque hora, ad quartam vicem, nisi prius supervenerit catharsis.

Six P.M.—The first pill produced copious evacuations; since which there have been no fits, but his manner is wild, and his conduct more extravagant than ever. The surface is cold and moist; the pulse rapid and small.

℞ Opii puri gr. xij.; Camphoræ ℈i. fiat massa, in pilulas sex dividenda. Sumat unam quarta quaque hora.

23d, eleven A.M.—Four pills have been taken, but there has been no sleep. He is much more quiet, however, and is evidently under the influence of the opium.—Omittantur.

Six P.M.—He has dozed a good deal during the day, and has been much less troublesome. Pupils dilated. His manner is subdued and childish.

Repet. Pil. Opii hora decubitus, et urgente delirio.

24th.—He has slept during the whole night, and is now perfectly collected. From this time all went on well.

The case seems rather interesting in a practical point of view. It was a well-marked instance of delirium tremens, but combined at its onset with such evident symptoms of cerebral congestion, that, had opium been at first administered, there is reason to suppose that the individual might have been *forced* into a state of apoplexy, or have become the subject of hopeless organic disease. I met a few months ago, in an Englishman, with a case very similar to the present, in which the same plan of treatment was pursued, and with a similar result. In general, when the head symptoms are not so prominent, on the bowels being thoroughly evacuated by the croton oil, (a medicine of immense value in such cases, and in all others when the object is to have a speedy and complete evacuation of the intestines,) I at once begin with opium.

---

*Inoculation of Hydrophobia.*—Some experiments have recently been made at the Veterinary College of Alfort upon this subject. A horse, two dogs, and three sheep, were inoculated with the saliva of a sheep affected with hydrophobia, at various stages of the disease, but no symptom of the malady was produced in the animals thus experimented upon. Four months had elapsed after their inoculation when this report was made.

---

*Ischuria successfully treated by cold Affusions.*—A dysenteric patient was affected by constant but ineffectual efforts to void his urine. He was placed in a warm bath, and bled from the arm, but the suppression of urine, which had existed forty-eight hours, was not relieved. Still continuing in the bath, Dr. Campbell directed cold water to be poured in a continued stream over the region of the bladder and pubis. In a short time the urine flowed in a full stream. The same treatment was continued the four following days. The patient was quickly restored to convalescence.—*North American Med. and Surg. Journal.*

---

*Eruption of Measles appearing only on one side of the Body.*—A child, three years old, had been observed never to perspire but on one side of the body. This singular anomaly had for some time ceased to present itself by the use of general bathing. The infant was afterwards attacked with measles, and the eruption only appeared on that side of the body which had before exhibited the greatest degree of vital activity. Recovery from the disease was not interrupted by any untoward symptom.—*Rust's Magazin.*

---

*Case of Aneurism of the Abdominal Aorta.* By CHARLES MAYO, Surgeon to the County Hospital in Winchester, &c.

A man, forty-eight years of age, had been subject, for six years, to violent attacks of pain in the back and loins, with numbness of the limbs, turbid urine, &c. These symptoms were more relieved by purgatives than any other remedies. Latterly, he sometimes complained of throbbing at the epigastrium, and for the last three weeks of his life had excruciating pains in the back and left inguinal region, which he compared to boiling lead pouring down the thigh. At this time, too, he kept the body constantly bent on the thighs. Bleeding, leeches, blisters, opiates, &c. were tried without avail, and he died exhausted by pain and irritation.

On examination post mortem, a large tumor was observed between the crura of the diaphragm, and stretching across the spine to the top of each kidney, especially the left. This tumor proved to be an aneurism of the aorta, into which this vessel opened by an aperture at its posterior part, an inch and a half in length, and half an inch in width. The left psoas muscle was softened, and its sheath filled with coagulated blood, extending into the groin. The state of this muscle, and the situation of the tumor, as connected with the kidneys, explain the principal symptoms observed during life.—*Provincial Medical Gazette.*

---

*Case of Bulimia, or Canine Appetite.* By Dr. PORTER, of Portsea.—This is a case of diabetes, in which the appetite was increased even to a greater than usual extent. It occurred in the person of a pale emaciated lad, aged nineteen, admitted July 31st, 1826, on board the Ragoon hospital convict ship. His skin was cold, his pulse feeble, and his belly large. At this time he devoured, solid and spoon victuals, twenty-six pounds, eight ounces; drink, twenty-two pounds, twelve ounces; while his excrements amounted to four pounds, eight ounces, and his urine to twenty-eight pounds. He was put on animal diet, and had opium administered in gradually increasing doses, till he took twelve grains daily. Under this treatment the appetite and urine gradually diminished, so that, on the 10th of January, 1827, they stand: food, three pounds, twelve ounces; drink, six pounds, fifteen ounces; the urine having diminished to seven pounds. On the 15th, it is stated that symptoms of pleurisy came on, and he died on the 20th.

On examination after death, the lungs were found highly inflamed, with effusion of lymph and serum. In the abdomen, the only appearance at all different from usual was, that the stomach and alimentary canal generally were pale and much more capacious than usual; and the same description applies to the kidneys. No disease existed in the brain or spinal marrow.—*Ibid.*

---

*Ossification of the Vitreous Humor.*—The eye alone, among the organs of sense, affords examples of ossification. Most frequently it is the choroid membrane which undergoes this change. In cataract, the degree of induration is hardly ever such as to warrant the appellation of bone. Haller assures us that he has seen the retina ossified, or at least that an ossific lamina occupied the place of this membrane. The same fact is noticed by Morgagni, Scarpa, Magendie, and Manoury. No genuine case of ossification of the vitreous humor has, however, been recorded. Lobstein,* indeed, says that ossifications of the hyatoid membrane are asserted to have occurred, but he appears to doubt the fact, and quotes no authority. Scarpa says that the hyatoid is sometimes opaque and thicker than natural. Morgagni speaks of it as occasionally cartilaginous. Beer mentions having found earthy matter in the interior of the vitreous humor, and occupying its place.

M. Krehn has lately met with a decided case of ossification of the vitreous humor. The preparation is placed in the Strasbourg Museum. It occurred in a man, seventy years old, who died of gastritis. The left eye was healthy, but the right presented the following appearances: The globe was evidently diminished in size; it had lost its spheroidal figure, and presented the appearance of four furrows or wrinkles, which corresponded with the insertion of the recti muscles. It was heavy, and felt hard. When a horizontal section was made from behind forwards, the sclerotic was found to be very thick, particularly at its posterior part, near the entrance of the optic nerve; the instrument was soon arrested by a hard body filling the whole space of the eyeball behind the chrystalline lens, and consequently occupying the place of the vitreous humor. Immediately within the sclerotic was the choroid membrane, distinct and rather thicker than natural. The retina was unchanged. The solid body within was marked by the same depression which had been observed externally. It was of a pale white colour, and was internally of a cellular texture, like the cancelli of the long bones. The crystalline was indurated, and of a yellowish white colour. The optic nerve was wasted.—*La Clinique.*

---

*Remarkable Predisposition to Hemorrhage.*—Dr. Schreyer, of Vogtsberg, states that, in a family of five children, under his observation, the eldest bit his tongue, and bled to death; the second and fourth are perfectly healthy; but the third and fifth have a remarkable tendency to hemorrhage. All these are of the male sex. The two above mentioned, one aged five years and the other fifteen months, have, at irregular periods, blue spots on the legs and thighs, which increase till they become as large as a pigeon's egg, when they assume a greenish blue colour: they do not bleed unless they are punctured; but, if this be done, the hemorrhage does not cease till the child faints, and the body is blanched. The blood which flows first is red, but after a time it becomes pale, like water in which flesh has been washed. Pressure with the

* Lobstein's Pathological Anatomy.

point of the finger, kept up for twenty-four hours, is sufficient, according to the testimony of the parents, to stop the bleeding. No coagulum ever forms, to plug up the vessels. Neither of the parents, nor their relatives, participate in this morbid condition; and it is remarkable that it has affected their children alternately, viz. the first, third, and fifth.—*Zeitschr. für Natur. und Heilkunde.*

---

*Habitual Hemorrhage from the Mammæ.*—S. A., æt. twenty-four, was admitted into the Konigsberg Hospital for this affection. Had been frequently attacked by epistaxis during her infancy; was married at the age of fourteen, the menstrual discharge not appearing until a year afterwards. At sixteen she became pregnant, the menses occurring at the regular interval during the two first months; they then ceased, but reappeared in the sixth and seventh months. She suckled her child (a boy) for two years, the menses appearing, and continuing to recur, from the second month after her delivery. On weaning her child, milk continued to be secreted in large quantity; and although, when the breasts became tense, it flowed from the nipple, yet, for her own comfort and relief from the distress it occasioned, she took the child of a neighbour, and continued to suckle it for a year and a half, and occasionally gave the breast to other children, the quantity of milk secreted was so great. She had now got to a period of four years after her confinement, when a practitioner, who was consulted, undertook to stop the excessive and continued secretion of milk, by repeated abstractions of blood; and this was performed seven times in the course of eight days. The flow of milk, upon this, ceased; but a more serious evil now took place; blood was discharged from both breasts, attended with much pain, and this became almost intolerable when the blood ceased to flow. This state had continued ever since, the blood coming away continually night and day, and also during the menstrual periods, but without affecting her health.

On her admission into the Konigsberg hospital, the patient had the appearance of a healthy well-fed woman, with something of a plethoric habit, and, with the exception of the affection for which she was admitted, and the attendant pain, in perfect health. The mammæ, which she stated to have been very large and full whilst the milk was secreted, but to have lost half their size since blood had been discharged, felt soft, and showed no evidence of inflammation. They were, however, extremely sensible to the touch, and she could not bear the pressure of her clothes upon them. From the nipples, which were of natural size and form, there trickled blood, sometimes of a bright red colour, sometimes thin and dark coloured, passing rapidly into putrefaction, and the quantity of which varied from three drachms to an ounce in the twenty-four hours. The blood could not be pressed from the breast as the milk had been. In cold weather especially there was much pain in the breasts, and, when the flow of blood stopped, the pains became intolerable, and extended to the neck and head, shoulders, and arms. She was free from fever, pulse slow and soft, skin dry, evacuations from the bowels and kidneys natural. During the progress of the case, the menses had continued to appear at the regular periods of four weeks, until a short time before the patient's admission, when, for the first time, they did not show themselves; whereupon a vicarious discharge of blood, apparently both from the lungs and stomach, took place.

Dr. JACOBSON had the patient ten weeks under his care, during which time various means were resorted to with a view to her relief. Leeches were repeatedly applied to the pudenda, and blood taken from the feet; digitalis, hydrocyanic acid, and alteratives, given internally; the semicupium and pediluvia employed, and a suspensorium mammæ applied. No alleviation was, however, obtained, and the difficulties of a cure seemed to be increased from the circumstance of discharge of blood from the lungs and stomach on the third appearance of her menses, (which usually continued eight days,) during her stay in the house. Unfortunately the patient most obstinately refused following the remedial means ordered for her, and she was on this account obliged to be discharged, so that the ultimate event of the case has not been ascertained.—*Rust's Magazin.*

---

*A Case of Exfoliation of the Cuticle.* Communicated by THOMAS NEWELL, M.D. Cheltenham, Surgeon Extraordinary to the King.

A young lady, when about twenty years of age, was first attacked with exfoliation of the cuticle from different parts of the body. The cuticle does not appear to undergo any change, nor does the cutis beneath seem to be otherwise altered than becoming inflamed; after which the points of connexion between it and the scarfskin become absorbed, or otherwise detached, so that this last drops off, and may sometimes be drawn from the hand like a glove. The attacks come on about twice a year, generally with considerable constitutional disturbance. Various remedies were tried without apparent benefit; but latterly she has been free from the complaint, since she went through repeated courses of the Cheltenham waters.

A case, of a nature similar to the above, was communicated to the Royal Society in 1769, by Mr. WARNER, of Guy's Hospital.

In the history of this case, it is mentioned that the fever which preceded the separation was of so peculiar a kind, that none of the medical men of great experience knew by what name to characterize it. The patient was very susceptible of alterations in the state of the air, and he had sometimes an attack twice in the course of the year. In this case the nails also separated, being gradually removed by the growth of new ones, a process which took five or six months, long after the skin had acquired a healthy state. This gentleman attributed his attacks to obstructed perspiration; but it seems more probable that this was the effect of the incipient stage of the disease, and not the cause of it.—*Midland Medical Reporter.*

---

### PRACTICAL MEDICINE.

*On the Utility of Camphor in Puerperal Mania.*—In the Journal der Praktischen Heilkunde, November 1828, several cases are related by Professor BERNDT, in which camphor was found very beneficial in this disease. In the cases related there was great sexual propensities, or even positive nymphomania. The evidence of the Professor is very substantial. Before he began the use of camphor, his success was unsatisfactory. In some cases the camphor was used in injections in the quantity of about ten grains, or it was administered internally in doses varying from one to four grains every hour, or less frequently in proportion to the urgency of the symptoms. Dr. Berndt had previously ascertained the inefficacy of the ordinary methods of cure, and the danger of narcotic medicines. He therefore resolved to try the effects of camphor, which appears to have been followed by the most favorable results.

---

*Utility of Borax in Diseases of the Skin.*—Dr. Reinhardt, of Mulhausen, states that he has cured several herpetic eruptions, of a scaly kind, with a watery solution of borax. He first tried the experiment upon himself. A solution of half a drachm of borax in an ounce of distilled water was applied over the back of both hands, which had been covered with the above species of eruptive disease. At first a sensation of heat and a redness of the part were produced. The lotion was discontinued for some days, and the use of it then resumed. The eruption gradually declined, and it had entirely vanished before the lotion was finished. In three similar cases the same remedy was employed with equal success. In one of these instances, a man, sixty years of age, had been afflicted with the disease for a considerable time.—*Hufeland, Journ. der Prakt. Heilkunde.*

---

*Union of Quinine and Digitalis in Phthisis Pulmonalis.*—Dr. Günther, of Cologne, first recommended these remedies in combination in 1825. He has recorded several cases in which the symptoms were decidedly relieved by this plan. One patient, a scrofulous girl, who was afflicted with tubercular consumption "au second degré," was completely restored to health. Each dose consists of two or three grains of the sulphate of quinine, and from one third to half a grain of powder of digitalis, with eight grains of fennel powder, four times a day.—*Ibid.*

---

*Intermittent Fevers treated by the external Application of the Sulphate of Quinine.*—In many cases it is impossible to administer particular medicines internally, either from the difficulty of swallowing them, from their producing vomiting, or from the insurmountable aversion or the tender age of the patient. It may also be added, that the secretions of the stomach very frequently interfere with the operation of remedies. From either of these causes we should be induced to try the endermic method of treatment. Dr. Speranza has thus employed the sulphate of quinine in a considerable number of cases of ague. He mentions, among others, fifteen cases of tertian fever, occurring in the spring, which immediately yielded to the quinine, which was placed upon a part denuded of the epidermis by the application of a blister. In each of these instances the fever had lasted many days; without any evident local derangement, excepting in two patients who had some gastric disturbance. Without prescribing any purgative remedies, a blister was first applied, and in most cases on the day of the paroxysm. The quinine was applied at the end of the fit, or at the beginning of the apyrexial period. The arm was usually selected as the most convenient part for the blister. The skin was first rubbed briskly with very strong vinegar, in order to hasten the vesication. Immediately after having raised the epidermis, eight or ten grains of the quinine, mixed up with simple ointment, were placed upon the abraded surface. The part was dressed on the second day, and both the remains of the ointment and the secretions from the surface were removed. About half the quinine was supposed to have been absorbed. By this mode of treatment, the fever at once disappeared in most instances. A second application of the remedy was not necessary.

Not only those fevers of which the type was originally tertian, but those which at first assumed a continued form and then became intermittent, were treated in the same manner with equal success. In no instance was there a relapse, which so frequently occurs after the internal use of the quinine. The application of the blister during the stage of fever produced no irritation of the urinary organs. In some individuals rather more inflammation of the blistered part occurred than is common, and in these instances topical emollients were necessary.—*Annali Universali.*

---

*Extract of a Letter respecting the anti-asthmatic Effects of the Tincture of Lobelia Inflata.*

"Having derived great benefit from the use of the tincture of Lobelia inflata, I am induced, in justice to its efficacy, and with the view of giving it publicity, to address you on its curative properties in my particular case. I have, for upwards of two years past, been afflicted with an inveterate asthma, which deprived me of natural rest, and the spasmodic effects of which were frequent and most distressing. When I found these paroxysms coming on, I took fifteen drops of the tincture, which invariably gave me infinite relief, although, previously to my using this remedy, the violent coughing fits often lasted from one to two hours. I should here remark, that I did not discover the virtue of the tincture of the Lobelia inflata until I had tried it about half-a-dozen times, in doses of fifteen drops each. Besides its medicinal virtue, it has, I find, the peculiar soothing quality of exciting expectoration without the pain of coughing. For myself, I have not the slightest doubt that, by continuing its use, I shall soon be restored to my wonted good health; and I feel assured that whoever makes trial of this excellent medicine will subscribe to the fidelity of this voluntary testimony of its merits.

"W. B. ANDREWS."

"4, *Henrietta street, Brunswick square.*"

N. B. The tincture is prepared according to the form published in the Medico-Chirurgical Review, by Mr. Snowdon, Haymarket.—*Med. Gazette.*

---

### MATERIA MEDICA.

*Adulteration of Sulphate of Quinine.*—This valuable medicine has been found adulterated with borax in a large proportion. The fraud is easily detected by throwing some alcohol over the powder, and then inflaming it. The flame will be of a green colour if any borax is combined with it.

---

### SURGERY.

*Strangulated Hernia reduced by the external Application of Belladonna.* By Dr. MAGLIARI.—A woman, æt. fifty, had been ruptured for several years. When Dr. M. saw the patient, all the symptoms of strangulation were present. They had existed twenty-four hours, and for their relief warm baths, leeches to the anus, poultices upon the tumor, &c. had been used. Castor oil had produced vomiting, which still continued. Dr. M. recommended the application of the following ointment: Lard four drachms, extract of belladonna, ten grains. The symptoms still continuing, the surgeon himself rubbed in the ointment a second time upon the hernial swelling. He used half the quantity above stated. On the following day, the vomiting had ceased; the tumor was diminished in size, but still the hernia was not completely reduced. The abdominal ring was, however, much dilated, and the intestine was not pressed upon. In a short time the reduction was completed.—*Revue Med.*

*Obstinate Cough cured by the Removal of a Portion of elongated Uvula.*—A woman had for more than a year been subject to the most violent paroxysms of coughing, which were sometimes so severe as to threaten suffocation. Various remedies had been applied without avail. Dr. PHYSICK examined the mouth, and ascertained that the cough depended upon a morbid elongation of the uvula. A portion of it was removed, and the cough entirely ceased.—*American Journal.*

---

*On Exfoliations from the Bones of the Pelvis, as causing the Obstinacy of Sinuses in this situation.* By JAMES SYME, Esq. Surgeon, and Lecturer on Surgery in Edinburgh.

The author of this paper, which appears to us highly interesting in a practical point of view, observes that obstinate sinuses are met with no where so frequently as in the region of the pelvis. Those which remain after the opening of abscesses depending on carious vertebræ, or caries of the hip-joint, are truly incurable, and, being unfortunately of common occurrence, have led to the opinion that little can be done for the remedy of any sinus so situated. The object of Mr. SYME is to show that the sinuses in question sometimes depend not on caries, but on death of bone, which exfoliating in some part of the pelvis far from the surface, causes continued irritation by the presence of the loose portion; whence it is proper, in the treatment of all sinuses in this part of the body not obviously proceeding from caries, to search for such exfoliations, and remove them if they are found to exist. Mr. Syme relates four cases in proof of the accuracy of his opinions, and the benefit of the practice he recommends.

In the first Case, a boy had a small fistulous opening in the upper and back part of the thigh, a little below the tuberosity of the ischium. The complaint commenced without any obvious cause about two years and a half ago, when a tumor, the size of an egg, made its appearance in the seat of the sinus. A surgeon evacuated by incision a great quantity of matter. The opening continued to discharge for a year, when a small bit of bone appeared at the orifice, and was removed. The sinus remained nearly well for six months, when the running again commenced, and continued for about a year. On introducing a probe, Mr. Syme detected a loose piece of bone, which was readily extracted by dilating the opening. The exfoliation appeared to have been detached from a spongy bone, probably the ischium. The boy quickly recovered.

CASE II.—A man, after severe exercise, had pain in the right hip. A collection of matter formed, and was let out by a surgeon. Some large abscesses afterwards formed in the thigh, lower down than the original one, and they were opened. The patient, finding that his complaint, although alleviated, was not cured, determined to leave it to nature. He permitted the disease to take its course for several years, occasionally working at his business as a cooper, when not suffering severely from pain. About two months before Mr. Syme saw him, he had again applied to a surgeon, by whom a piece of bone was removed, which was found sticking at the orifice of a sinus. It was ascertained that there was more bone to come away, and attempts were made to dilate the opening by sponge tent, which much aggravated the sufferings of the patient. He now applied to Mr. Syme, who found a large diffused abscess occupying the upper and back part of the thigh, and extending from the hip half-way to the knee. In the fold which lies between the hip and thigh, there was an opening which allowed the probe to enter fully three inches in the direction of the tuberosity of the ischium, and at the bottom of this passage a loose piece of bone was felt. The man was now pale, emaciated, and desponding. Mr. S. opened the abscess, and let out several ounces of pus. On the following day the sinus was dilated to the bottom, so as to admit a finger, and it was found that the exfoliation lay in a cavity between the origins of the flexor muscles of the knee. The mouth of this cavity was dilated, and a piece of bone, about half the size of a sixpence, was easily extracted. In the course of two or three days the patient walked a mile, and at the end of two or three weeks he was able to resume his occupation. Some months afterwards another small piece of bone was removed, and the man remained free from complaint.

Two similar cases are related by Mr. Syme, which illustrate the same pathological principle, and good effects of his practice.

Mr. S. trusts that the history of these cases will excite a more discriminating diagnosis and active treatment of sinuses of the pelvis. It is not his intention to dwell at any length at present upon the origin of the exfoliations. It appears evident, he remarks, that they cannot result from the direct effects of violence; since, in all the cases detailed, the bone concerned was securely protected by its situation from any such injury. In all of them, except the first, where no information could be obtained as to the origin of the complaint, there was violent muscular contraction; and Mr. S. is inclined to think that this was the exciting cause of inflammation and death of the bone. The subject is curious and worthy of investigation, but of little importance when compared with the practical benefit which may result from a knowledge of the fact that sinuses of the pelvis sometimes depend on loose exfoliations, which will not find their way out unassisted, but which may be readily removed artificially, with the effect of a speedy and perfect cure.—*Edinburgh Med. and Surg. Journal*, April.

---

*Case of Strangulated Hernia, in which six Inches of the Intestine were removed.* By John Simpson, M.D. Bath.

A man, about sixty years of age, had long been troubled with a large inguinal hernia, which had frequently descended into the scrotum. In the year 1816, four days before Dr. Simpson was sent for, it had come down, and could not be replaced ; it then became strangulated. Various and judicious measures had been adopted by a gentleman in his neighbourhood, but it became necessary to perform an operation. On opening the sac, a large quantity of fluid escaped ; omentum and intestine were contained in it, and both were in a state of mortification. The stricture, which was at the inner ring, was divided, and great relief thus given; but the parts were glued together by old adhesions, owing to which the bowel could not be returned. Next day, the symptoms continuing urgent, an incision was made along the protruded intestine, when a very large quantity of black feculent matter was discharged. The following day he was better; and the omentum, and six or seven inches of intestine, supposed to be ileum, were removed with the knife: very little bleeding occurred. A pad was applied over the upper part of the wound, which was removed occasionally, so as to empty the bowels. In about three weeks some feces passed per rectum, and in a few months the artificial anus entirely healed. Four years after he was alive and well.—*Midland Medical Reporter.*

---

*Chlorides of Soda and Lime in Sloughing Sores.* (From "Cases illustrative of the beneficial Effects of the Chlorurets of the Oxides of Sodium, and of Calcium, of the Chevalier LABARRAQUE, of Paris. By J. G. F. HASSELL, M.D. late of the British Army.")

In January, 1827, a young woman, named Josephine, aged twenty-five, of a sanguine temperament, was admitted into the Boulogne Civil and Military Hospital, which is superintended by two experienced medical practitioners, namely, Messrs. GORÉE and ROUXEL, both doctors of medicine and surgery. This patient was afflicted with a sarcomatous tumor, the size of a large orange, situated on the inside of her left thigh. Dr. Gorée proposed the removal of this tumor by a surgical operation, to which the patient not submitting, she was soon discharged.

A few weeks after having quitted the hospital, she received, by accident, a blow on this tumor, with which she had been afflicted for upwards of a year. After this accidental excitement, the tumor inflamed violently, so much so that the swelling extended over the whole thigh and knee, causing great muscular contraction and deformity of the limb. This induced the patient to apply for readmission into the hospital, where she was again received in the beginning of March. Independent of a proper medical treatment, the tumor was poulticed for several days, after which suppuration took place: a dark-coloured fetid matter escaped through a small aperture on the lower part of the tumor, about two inches on the inside above the knee; but this natural opening (caused by an increased action in these parts) soon degenerated into a malignant and sloughing ulcer, which made a rapid progress both in the adjacent integuments and deep-seated muscles.

On the 12th of March, Dr. Gorée made a large longitudinal incision through the diseased integuments and fascia, out of which more than two pounds of fetid matter were discharged. The smell of this corrupted ulcer was the most offensive that I ever experienced in practice. The cavity of the wound presented a most frightful and alarming aspect. It was fomented with a mixture of one part of the chloruret of sodium* and twelve parts of tepid water, by which the putrid smell was subdued in a few seconds, to the great surprise of the attendants and patients present in the sick ward. The whole cavity of this enormous wound was filled up with fine dry soft French lint, over which two or three ounces of the concentrated chloruret of sodium were sprinkled; a large thick compress and bandage completed the dressing, which was saturated with the diluted mixture of the chloruret in the proportions as above mentioned. On the following morning the offensive odour of the wound was considerably lessened, and a vast deal of fetid matter was again discharged. To the dressing adhered not only the sloughy integuments, but also a large mass of disorganized cellular substance, muscular fibres, and veins, which came

* The chloruret of the oxide of sodium of the French chemists is the chloride of soda of the British.—EDITORS.

away without much resistance, by hooking it out with the assistance of the finger. This corrupted fleshy mass weighed upwards of a pound. The wound was then treated as before, and the same plan was pursued for several successive days until the fetid matter was discharged, and the putrid smell entirely subdued. By this treatment the wound was converted into a simple and healthy one about the 18th of March; a rapid and healthy granulation soon filled up its extensive cavities; the patient's exhausted frame was likewise gradually recruited; and, by paying attention to the digestive organs, and prescribing a light but nourishing diet, she soon recovered her strength and general health.

Dr. Hassell relates another case, that of a gentleman, of full habit, aged sixty, whose constitution had been much impaired by luxury, and who had a very painful carbuncle in his neck, which a practitioner had mistaken for a common boil, and directed to be poulticed. The Doctor was consulted, and found seven or eight sinuses in the carbuncle, "through which a dark yellow, greenish, bloody, and irritating ichor issued:" in less than twenty-four hours afterwards, the inflammation had extended upwards to the occiput, and downwards over all the cervical vertebræ and both scapulæ; an extensive sloughing of the integuments followed. The patient being in imminent danger, Dr. H. made several deep incisions through the integuments; and, after a considerable quantity of dark blood and fetid matter had escaped, fomented the parts with equal proportions of the infusion of poppies and Labarraque's liquid. The wounds were filled up with lint dipped in the same. A mixture and an opiate draught, containing also fifteen drops of the liquid, were given the patient. The next day the gangrenous parts came away with the dressings; and, these being five or six times renewed, a few emollient poultices completed the cure.

---

M. Dupuytren's *Treatment of Spots on the Cornea.*—Patients have flocked to the Hôtel Dieu for some years for the treatment of spots on the cornea, as formerly under Desault, for that of chronic ophthalmia of a scrofulous or other nature.

The treatment employed by M. Dupuytren is as follows:

A bleeding, if there be violent irritation. Leeches to the temples, if this irritation is less. Afterwards one or two mild purgatives, two or three days intervening between each. After which a seton made of cotton threads, united in a cylinder, and some inches in extent, is introduced under the skin at the back of the neck. In fine, the insufflation, or blowing into the eye or eyes, with the barrel of a quill, (the eyelids being separated,) a pinch of an impalpable powder, composed of ℞ Oxyd. Zinci impur., Sacchari Crystal., Hydr. Submuriatis, āā partes æquales. Misce, fiat pulv. subtilissim. The size of the pinch may vary, and the insufflation should be repeated night and morning. The patients ought neither to wash nor dry their eyes after it.

When there is no disease on the eyelids, no inflammation, no irritation of the conjunctiva, the insufflation of the above powder generally suffices to remove the spots. Those which are recent and slight are completely dissipated in a few weeks by this treatment. The thicker and larger patches are ordinarily cured in a month or six weeks; and very frequently patches which occupy nearly the whole of the cornea, and completely cover the pupil, entirely intercepting the passage of light into the eye, disappear entirely in a few months. —*Ratier's Medical Guide to Paris.*

M. Dupuytren's *Treatment of Phagedenic and Corroding Herpes.*—There is no physician who has not had an opportunity of observing and treating phagedenic or corroding herpes, and experienced a disagreeable proof of the inefficacy of the anti-herpetic, anti-scrofulous, anti-venereal remedies, and others, which have been tried by turns against this cruel disease, according to its different appearances and its supposed nature. We know that, in spite of all the remedies, the phagedenic herpes eats and destroys the nose, the lips, the cheeks, the eyelids, the ears, the temples; parts which it more especially and frequently attacks. Fire itself seems to irritate, as well as arsenical paste: these agents have, besides, the inconvenience of destroying the parts on which they are applied, and to add to their deformity. These motives have for a long time induced M. Dupuytren to seek other remedies against phagedenic herpes, and it seems certain that they may be cured without deformity, by the use of the following powder:

| | | |
|---|---|---|
| ℞ Hydrarg. Submur. præcip. partes | ........... | 199 |
| Oxidi Arsenici albi, vel Acidi Arseniosi | partem............. | 1 |
| | | 200 |

This remedy, which acts rather as a specific than as a caustic, may be variously employed. If the surface of the herpes is ulcerated, moist and cleaned, it is powdered with a little puff, charged with the above-described powder, so as to cover it with a thick layer of about the twentieth part of an inch. If this surface is covered with a scab, it must be thrown off by means of a poultice, and then it is dusted as has been just described. In fine, if the herpes is actually covered with an imperfect cicatrix, it must be destroyed; twenty-four hours after, the surface is dusted, when it must necessarily have ceased bleeding.—*Ibid.*

### MIDWIFERY.

*Expulsion of the Placenta four Months after Delivery.*—A woman was delivered in January of a dead child, in which putrefaction had commenced in different parts of the body. The midwife made many useless efforts to extract the placenta: she pulled so hard, indeed, by the funis as to break it off. The placenta still remained in the uterus. The cervix uteri closed, and neither uterine pains nor any discharge indicated the probability of the expulsion of the afterbirth. The woman enjoyed a perfect state of health till the following May: slight pains and a sanguineous discharge then appeared. These symptoms lasted but a short time, and again returned. They were now more severe, and were followed by the expulsion of the placenta, the presence of which in the uterus, during so long a period, had been productive of no inconvenience.—*Gemein Deutsche Zeitschr. für Geburtskunde.*

*Pregnancy, with Cancer of the Cervix Uteri.*—Dr. Laubreis, a practitioner in Bavaria, has related two cases of this nature: the first proves that conception may take place, if the full term of utero-gestation be completed, notwithstanding the presence of carcinoma of the neck of the uterus, provided it be not far advanced. In the second case, the scirrhus was far in the ulcerative stage before impregnation took place, and the woman miscarried at the end of the third month, and died, by which an opportunity was afforded of examining the parts.—*Journal für Geburtshülfe, &c.*

### CHEMISTRY.

*Method of detecting Arsenic in Sulphur.*—MM. Geiger and Reimann say that the presence of arsenic in sulphur, to the amount only of 0.000061, may be discovered in the following manner: A certain quantity of precipitated sulphur, flowers of sulphur, or ordinary sulphur finely pulverised, is to be digested with ammonia for a considerable time, then filtered, and afterwards the clear liquid acted upon by excess of muriatic acid. If a yellow precipitate occurs, it is an indication of the presence of arsenic; if not, the liquid is to be evaporated until only a few drops remain. A little ammonia is then to be added; afterwards a small quantity of muriatic acid; and, finally, a little solution of sulphuretted hydrogen. If there be the smallest quantity of arsenic, it will be rendered evident by a yellow precipitate.—*Bull. Univ.* (*Quarterly Journal of Science.*)

### MISCELLANEOUS.

*On Feigned Diseases of the Heart.* By Dr. Quarrier.—A man, named Chapman, became notorious in the Royal Marine Artillery for possessing powders capable of producing symptoms so closely resembling those of diseased heart, as to deceive the medical attendants, and lead to the men procuring exemption from duty. These powders, it appears, consisted chiefly of the Helleborus albus; and the drug was actually administered in the immense doses of one drachm or more, when it was intended to produce a very decided effect.—*Provincial Medical Gazette.*

